# *wrt-2* expression oscillates during larval development

**DOI:** 10.17912/4bw7-ep56

**Published:** 2018-10-11

**Authors:** Ana G Vera-Cruz, Siavash Amon, Sabih Rashid, Lesley T MacNeil

**Affiliations:** 1 Department of Biochemistry and Biomedical Sciences, McMaster University, Hamilton, ON, Canada.; 2 Farncombe Family Digestive Health Research Institute, McMaster University, Hamilton, ON, Canada.; 3 Michael G. DeGroote Institute for Infectious Disease Research, McMaster University, Hamilton, ON, Canada.

**Figure 1.**
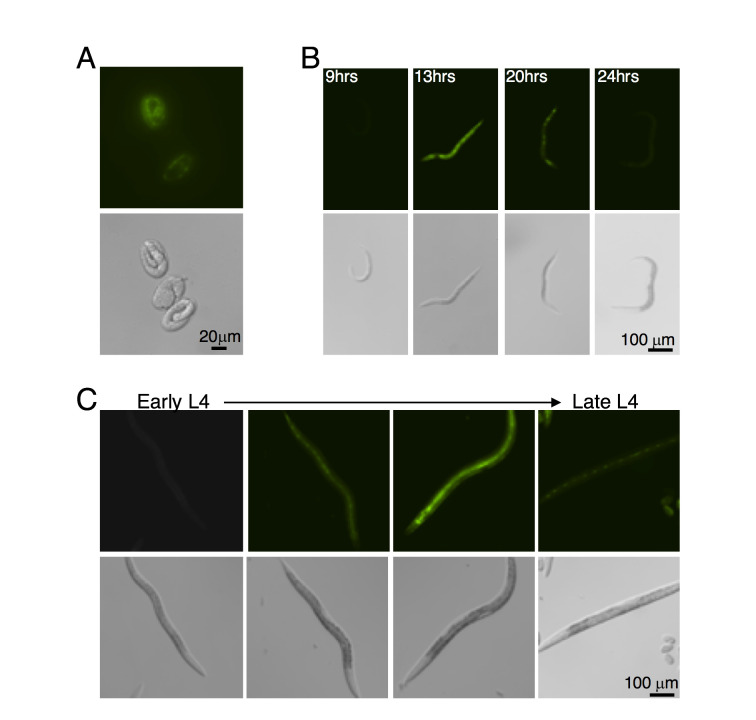
*Pwrt-2::GFP-pest* expression oscillates throughout development. A. *Pwrt-2::GFP-pest* expression is first observed late in embryogenesis but is turned off in L1 arrested animals. B. Time course of GFP expression from L1 arrest (20°C). C. Expression of *Pwrt-2::GFP-pest* during L4. GFP is not detected in early L4, is observed mid-late L4, and is turned off before the adult stage.

## Description

*wrt-2* encodes a hedgehog-like protein that is weakly required for molting (Kouns et al., 2011). As monitored using a transcriptional GFP reporter, *wrt-2* is expressed in seam and hypodermal cells throughout development (Apock et al., 1999). However, *wrt-2* transcript levels oscillate with a period consistent with the timing of the molting cycle (Hendriks et al., 2014). Because standard GFP reporters do not capture rapid changes in gene expression, we generated a *wrt-2* transcriptional reporter driving expression of a rapidly degraded GFP (GFP-pest) to observe the precise timing of *wrt-2* expression throughout development. Consistent with previous findings, *Pwrt-2::GFP-pest* expression was observed in hypodermal and seam cells at all larval stages. However, expression was not constant throughout development but rather cycled on and off at each larval stage (Figure 1 B and C). *Pwrt-2::GFP-pest* expression was first observed in the embryo and turned off at hatching (Figure 1A). Expression ceases at the end of L4 and no expression was observed in adult animals. We confirm that *wrt-2* expression oscillates with the molting cycle and demonstrate that these oscillations are driven by the 5’ regulatory region of *wrt-2*.

## Methods

Eggs were collected by hypochlorite treatment, washed, and incubated for 18hrs in M9 buffer to achieve L1 synchronization. Following L1 arrest, animals were propagated at 20°C on NGM agar seeded with *E. coli*OP50. GFP expression was observed in a population of approximately 50 animals over time and individual animals representative of the population were mounted and photographed. For L4 animals, vulval morphology was also used to verify the relative age of animals in Figure 1C.

## Reagents

Strain:

N2 animals were microinjected with a mix containing 50ng/mL *Pwrt-2::GFP-pest* and 40ng/mL *Pabu-11::mCherry-pest.* *Pabu-11::mcherry-pest* was used as a co-injection marker because *abu-11* transcript levels oscillate during development with timing opposite to that of *wrt-2* (Hendriks et al., 2004). We expected that this reporter would be expressed at times when *Pwrt-2::GFP-pest* was not visible. However, at this dose, *Pabu-11::mCherry-pest* is visible in the pharynx at all larval time points and in the adult. The transgene was integrated by UV treatment and outcrossed with N2 for 10 generations to generate LMN17*(ltmIs1[Pwrt-2::GFP-pest::unc-54-3’UTR + Pabu-11::mCherry-pest::unc-54-3’UTR]).*

Constructs used:

A fragment of 1307 bps, corresponding to the region immediately upstream the *wrt-2* start codon, was PCR amplified from genomic DNA and cloned into the SalI and BamHI sites in pCMH1225 (Perales et al., 2014). The *abu-11* promoter, representing 1823bp of sequence upstream of the start ATG, was cloned into the SalI and BamHI sites of pCMH1183 (Perales et al., 2014).
